# Crosstalk between ubiquitin ligases and ncRNAs drives cardiovascular disease progression

**DOI:** 10.3389/fimmu.2024.1335519

**Published:** 2024-03-07

**Authors:** Jia-Rui You, Zeng-Jin Wen, Jia-Wei Tian, Xiao-Bing Lv, Rong Li, Shu-Ping Li, Hui Xin, Pei-Feng Li, Yin-Feng Zhang, Rui Zhang

**Affiliations:** ^1^ Department of Cardiology, The Affiliated Hospital of Qingdao University, Qingdao University, Qingdao, Shandong, China; ^2^ Institute for Translational Medicine, The Affiliated Hospital of Qingdao University, College of Medicine, Qingdao University, Qingdao, China; ^3^ Department of Emergency Internal Medicine, The Affiliated Hospital of Qingdao University, Qingdao, Shandong, China; ^4^ Department of Cardiology, The Affiliated Qingdao Third People’s Hospital of Qingdao University, Qingdao University, Qingdao, Shandong, China

**Keywords:** miRNA, lncRNA, circRNA, ubiquitination, deubiquitination, UPS

## Abstract

Cardiovascular diseases (CVDs) are multifactorial chronic diseases and have the highest rates of morbidity and mortality worldwide. The ubiquitin–proteasome system (UPS) plays a crucial role in posttranslational modification and quality control of proteins, maintaining intracellular homeostasis via degradation of misfolded, short-lived, or nonfunctional regulatory proteins. Noncoding RNAs (ncRNAs, such as microRNAs, long noncoding RNAs, circular RNAs and small interfering RNAs) serve as epigenetic factors and directly or indirectly participate in various physiological and pathological processes. NcRNAs that regulate ubiquitination or are regulated by the UPS are involved in the execution of target protein stability. The cross-linked relationship between the UPS, ncRNAs and CVDs has drawn researchers’ attention. Herein, we provide an update on recent developments and perspectives on how the crosstalk of the UPS and ncRNAs affects the pathological mechanisms of CVDs, particularly myocardial ischemia/reperfusion injury, myocardial infarction, cardiomyopathy, heart failure, atherosclerosis, hypertension, and ischemic stroke. In addition, we further envision that RNA interference or ncRNA mimics or inhibitors targeting the UPS can potentially be used as therapeutic tools and strategies.

## Graphical Abstract

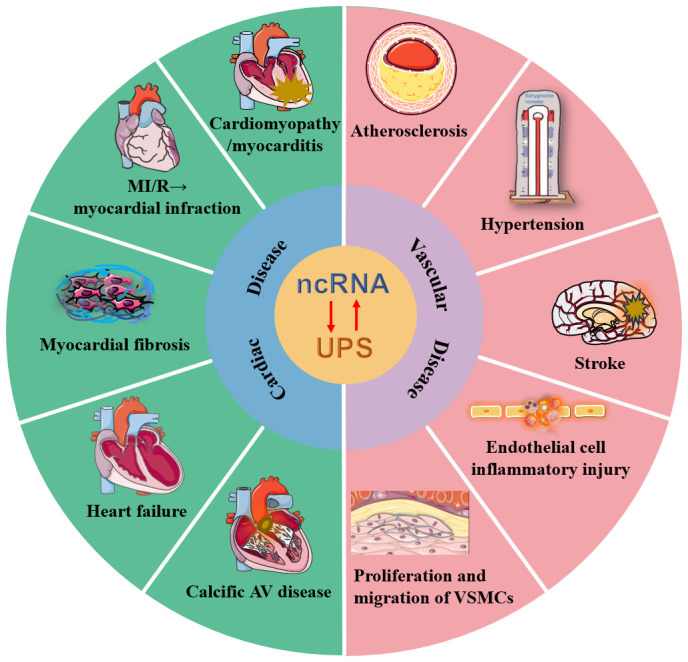

## 1 Introduction

Cardiovascular diseases (CVDs) are a wide spectrum of disorders affecting hearts or vessels, including myocardial ischemia/reperfusion (I/R) injury, myocardial infarction (MI), cardiomyopathy, myocarditis, heart failure (HF), atherosclerosis, hypertension (HT), and ischemic stroke (1–4). Myocardial ischemia and MI can induce excessive ventricular remodeling and the development of cardiomyocyte hypertrophy, leading to HF (2, 3, 5). Myocarditis is an inflammatory disease that damages the tissue of the heart muscle, and the main reasons are autoimmune response, viral infections, or immune reactions after infection (5). In addition, vascular diseases are progressive inflammatory pathologies, that can result in endothelial injury, atherosclerotic plaque formation, and HT, especially pulmonary arterial hypertension (PAH) (6).

The ubiquitin–proteasome system (UPS) is the main nonlysosomal pathway for targeted protein degradation in eukaryotic cells (7–9). The UPS consists of three components: a ubiquitination mechanism (ubiquitin, E1, E2 and E3 enzymes), deubiquitinating enzymes and the 26S proteasome (10). First, E1 activates the C-terminal glycine residues of ubiquitin by using ATP, and then activated ubiquitin is transferred to E2 by a high-energy thioester bond linking the C-terminal carboxyl group of ubiquitin to the active cysteine in E2. Third, E3 catalyzes the covalent attachment of ubiquitin to the lysine side chain of the substrate protein (11, 12). Subsequently, substrate proteins modified by polyubiquitin chains are degraded via the 26S proteasome. The 26S proteasome consists of two 19S regulatory complexes and one 20S complex (13, 14). E3 ubiquitin ligases regulate various aspects of eukaryotic biology by promoting ubiquitination degradation of target proteins (15). For example, smad ubiquitin regulatory factors (Smurfs) come from the HECT family of E3 ubiquitin ligases and consist of two members, Smurf1 and Smurf2 (16). Additionally, ubiquitination is invertible, and deubiquitylation can remove ubiquitin from specific substrate proteins by deubiquitinating enzymes, which maintains ubiquitin system homeostasis (10, 17, 18). The ubiquitin-specific protease (USP) family, which includes USP3, USP7, USP14, and USP28, is one of the major deubiquitinating enzymes (19). Encouragingly, both ubiquitination and deubiquitinating enzymes, such as Smurf1, Smurf2, carboxyl terminus Hsp70-interacting protein (CHIP), Nedd4, WWP2, and YOD1, are involved in the regulation of cardiovascular function and may allow for new targeted therapeutic opportunities (7). Notably, the UPS plays an essential role in many aspects of biological processes, such as cell cycle control, cell differentiation and survival, protein turnover, protein quality control and signal transduction, some of which involve ncRNAs (7–9).

Noncoding RNAs (ncRNAs), such as microRNAs (miRNAs), long noncoding RNAs (lncRNAs), circular RNAs (circRNAs) and small interfering RNAs (siRNAs) are an increasing spectrum of transcriptional regulatory factors with no coding potential but significant structural and regulatory functions (20–22). MiRNAs are single-stranded RNAs consisting of 20-22 nucleotides, and their primary function is to combine complementary sequences on mRNAs to repress their translation (5). LncRNAs are a class of ncRNAs that are more than 200 nucleotides in length and can regulate the expression of specific miRNAs and consequently modulate miRNA downstream target genes (23, 24). Long intergenic noncoding RNAs (lincRNAs) function as essential regulatory factors of gene expression (25). CircRNAs are stable single-stranded ncRNAs with closed-loop structures with miRNA sponges that interact with RNA-binding proteins as the main molecular mechanism (26, 27). Moreover, siRNAs mediated by RNA interference (RNAi) are significantly versatile tools to target corresponding mRNA and silence protein-coding genes and have been used clinically for tissue targeting based on complex chemical modifications and carrier/ligand coupling (28). One of the roles of ncRNAs is the precise control of protein stability via UPS.

There are emerging reviews about the interaction of ncRNAs and UPS on cancer, especially non-small cell lung cancer, hepatocellular carcinoma, colorectal cancer, and gastric cancer (29–31), while implications of protein ubiquitination modulated by ncRNAs in cardiovascular aspects have not been systematically elaborated and comprehensively summarized although multiple studies have demonstrated that ncRNAs have a profound impact on the genetic and proteinic programming of CVDs through their interaction with the UPS (32). Fine-regulation between ncRNAs and the UPS is critical in the epigenetics of CVDs. Overall, this review paper emphasizes the mechanisms of ubiquitination in the context of the widespread association of ncRNAs with CVDs and attempts to elucidate the perturbations of the crosstalk between the UPS, especially ubiquitin ligases, and ncRNAs in CVDs. Overall, we provide the latest advances and look forward to future cardiovascular treatment strategies targeting ncRNAs and ubiquitin ligases.

## 2 Crosstalk between ncRNAs and the UPS

**Figure 1:**
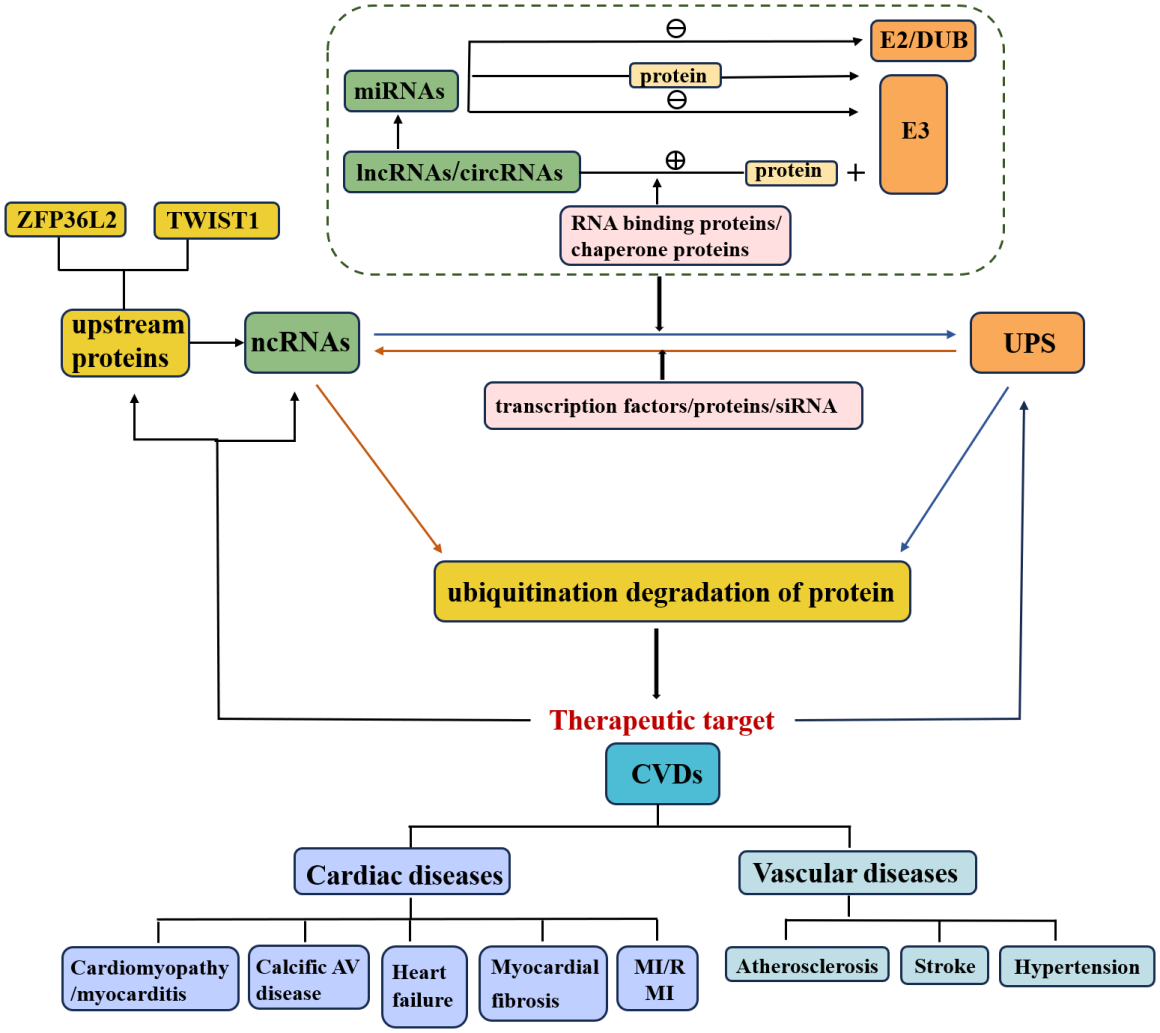
Relationship of ncRNAs, UPS and CVDs. The crosstalk of the UPS and ncRNAs affects the pathological mechanisms of CVDs. NcRNAs that regulate ubiquitination or are regulated by the UPS are involved in the execution of target protein stability.

In recent years, multiple crosstalk between ncRNAs and the UPS has been demonstrated to play essential regulatory roles in various diseases, particularly CVDs (**Figure 1**) (33). First, the positive or negative regulation of miRNA on ubiquitination has gradually attracted considerable research attention. (1) MiRNAs directly inhibit E3 ubiquitin ligase expression and subsequently suppress ubiquitination and degradation of target proteins. (2) MiRNAs directly inhibit ubiquitin-conjugating enzyme (E2) expression. (3) MiRNAs directly inhibit the expression of deubiquitinating enzymes and disrupt the ubiquitination balance of deubiquitinating enzymes and target proteins. (4) MiRNAs regulate multiple UPS components. (5) MiRNAs target some proteins that are involved in the regulation of E3 ubiquitin ligases.

Furthermore, the study of upstream regulatory pathways of miRNAs, such as lncRNAs and circRNAs, is also a meaningful research direction (29, 31). (1) LncRNAs/circRNAs promote the ubiquitination and degradation of target proteins. a) LncRNAs/circRNAs interact with target proteins and promote their ubiquitination and degradation. b) LncRNAs/circRNAs enhance the interaction between the E3 ubiquitin ligase and its target. c) LncRNAs combine with RNA binding proteins or chaperone proteins that are involved in the interaction between the E3 ubiquitin ligase and target protein (20). (2) LncRNAs inhibit the ubiquitination and degradation of target proteins. (3) LncRNAs/circRNAs act as competitive endogenous RNAs (ceRNAs) for miRNAs and thus regulate the expression of E3 ubiquitin ligases/deubiquitinating enzymes.

**Figure 2:**
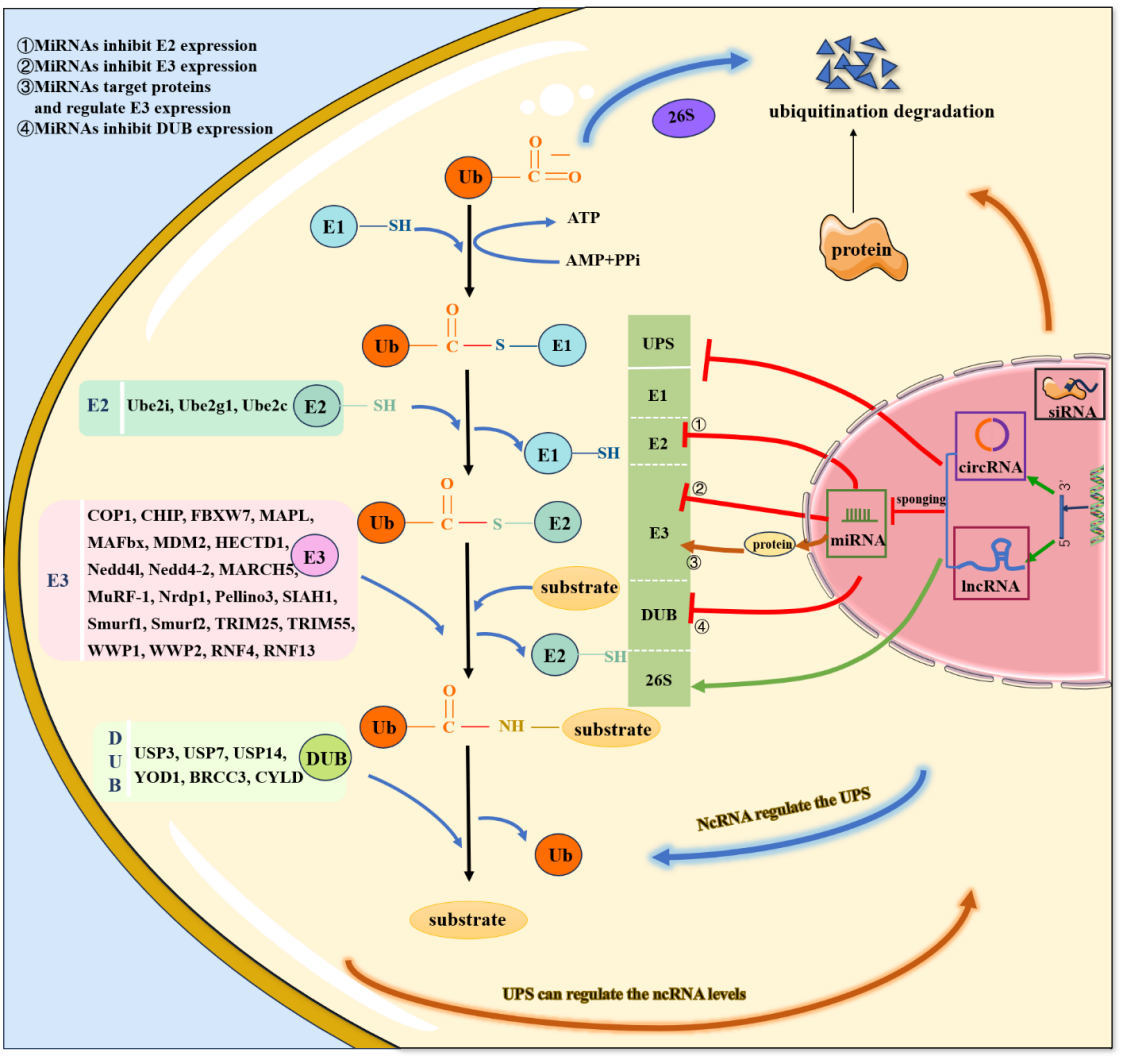
Crosstalk of ncRNAs and UPS.

In addition to being regulated by ncRNAs, the UPS can also regulate ncRNA levels. On the one hand, E3 ubiquitin ligases directly regulate ncRNAs (**Figure 2**), thereby promoting ubiquitination and degradation of target proteins. On the other hand, the UPS indirectly regulates ncRNAs through the involvement of transcription factors/proteins. Specifically, knockdown of the E3 ubiquitin ligase by siRNA has been indicated to be involved in the pathology of CVDs via regulation of downstream protein levels and could serve as a potential therapeutic strategy.

## 3 Crosstalk between ubiquitin ligases and ncRNAs drives cardiac disease progression

### 3.1 Apoptosis and myocardial infarction

Ischemic conditions during myocardial I/R cause anoxic damage to cardiomyocytes, reducing mitochondrial ATP synthesis via oxidative phosphorylation, altering cell membrane permeability, and finally inducing apoptosis; therefore, timely restoration of blood flow is abstractly crucial for alleviating acute MI (34). Untimely reperfusion can produce further damage and exacerbate cardiac dysfunction, which is referred to as myocardial I/R injury, anoxia/reoxygenation (A/R) injury or hypoxia/reoxygenation (H/R) injury (34, 35).

**Figure 3:**
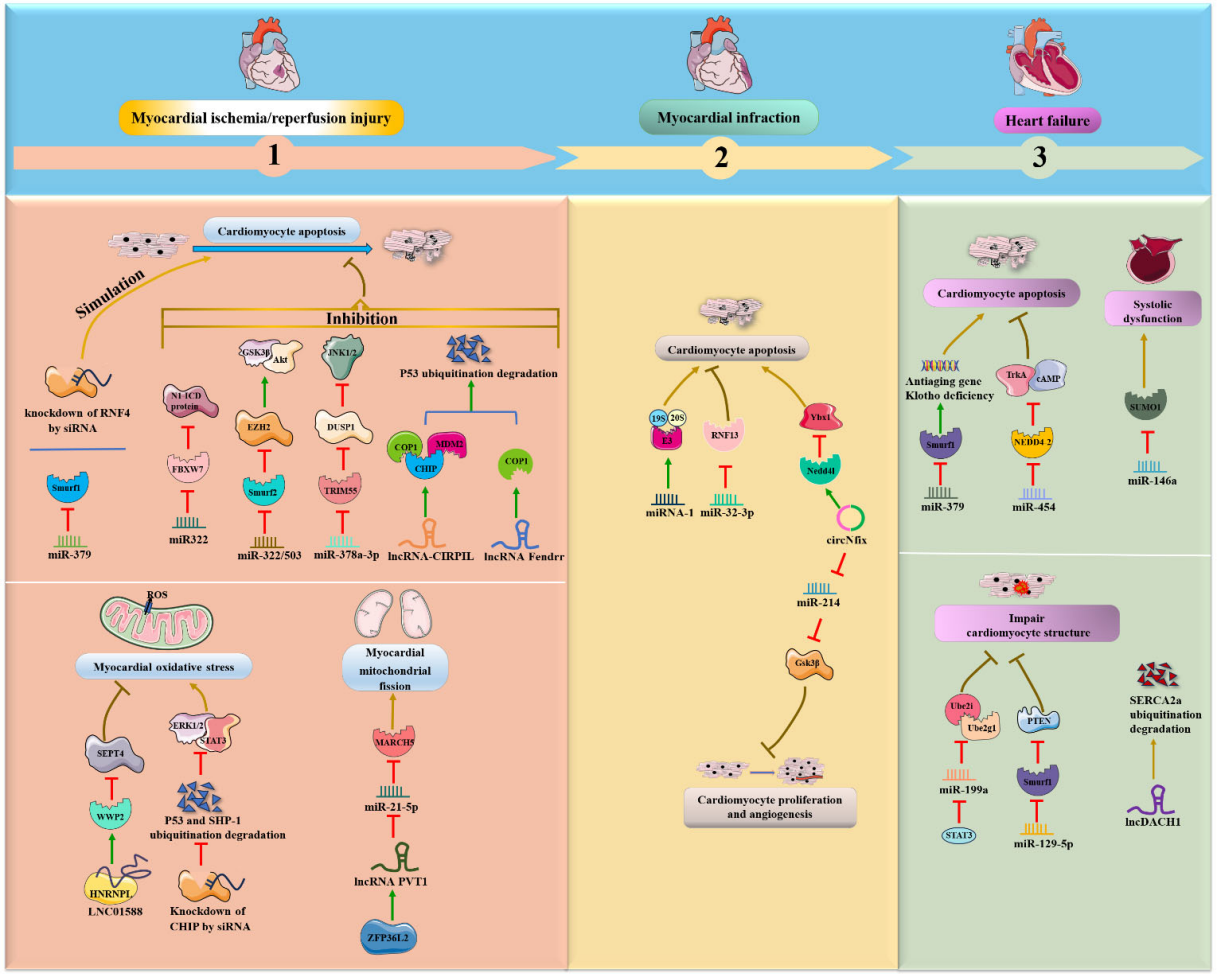
NcRNAs and ubiquitination in cardiac diseases. The crosstalk of ncRNAs and ubiquitination regulates cardiac diseases, such as MI/R, MI, myocardial. NcRNAs and ubiquitination are involved in cardiac diseases by regulating target protein expression.

Some miRNAs, particularly miRNA-424(322) and miR-378a-3p, were shown to be significantly decreased in myocardial I/R mice or rats, while overexpression of these miRNAs improved myocardial I/R-induced apoptosis and injury by inhibiting ubiquitin ligase (FBXW7, Smurf2 and TRIM55) expression and downstream protein degradation (**Figure 3**). In detail, Chen, Dong et al. (36) found that enhanced expression of miR322 prevented reperfusion-induced cardiomyocyte apoptosis by reducing the expression of the ubiquitin ligase FBXW7 and increasing N1-ICD protein in the hearts of mice *in vivo*. Similarly, miR-322/503 directly binds to and inhibits the translation of Smurf2 (an E3 ubiquitin ligase), thereby suppressing Smurf2 ubiquitination-mediated EZH2 degradation and activating the Akt/GSK3β pathway, ultimately reducing myocardial apoptosis and infarct size in the I/R rat model *in vivo* and *in vitro* (37). In addition, miR-378a-3p suppresses ubiquitinated degradation of DUSP1 by decreasing expression of the E3 ubiquitin ligase TRIM55 and then inactivates JNK1/2, eventually preventing myocardial I/R-related apoptosis and injury in rat myocardial I/R models *in vivo* (38). Conversely, miR-379 could facilitate cardiomyocyte apoptosis by inhibiting Smurf1 expression; meaningfully, exogenous Smurf1 protein could rescue miR379-induced apoptosis in cardiomyocytes (39). Additionally, circUSP39 promotes cardiomyocyte apoptosis and accelerates H/R-induced injury by sponging miR-362-3p and upregulating TRAF3 expression (40).

Interestingly, lncRNAs are also emerging as an essential class of regulators in the pathology of myocardial I/R. Collectively, lncRNA-CIRPIL, lncRNA Fendrr and LINC01588 were shown to be downregulated in myocardial I/R mice or rats and to function as negative mediators of myocardial I/R-induced cardiomyocyte apoptosis by enhancing E3 ubiquitin ligases (COP1, CHIP, MDM2 and WWP2) *in vivo*. First, lncRNA-CIRPIL interacts with p53 and sequesters it in the cytoplasm, directly accelerating the ubiquitin-regulated degradation of p53 by upregulating the expression of E3 ubiquitin ligases COP1, CHIP and MDM2, thereby attenuating myocardial A/R-induced apoptosis and injury (41). Similar to lncRNA-CIRPIL, lncRNA Fendrr suppresses myocardial H/R-induced apoptosis by facilitating the ubiquitination degradation of p53 via COP1 (42). Moreover, LNC01588 can make direct contact with the HNRNPL protein and upregulate the E3 ubiquitin ligase WWP2, which then reduces the levels of downstream SEPT4, thereby ameliorating myocardial oxidative stress and MI during myocardial I/R damage (43). On the other hand, inhibition of p53 ubiquitination degradation and inhibition of WWP2 attenuate the protective effects of lncCIRPIL and LINC01588 (41, 43). Conversely, the ZFP36L2- lncRNA PVT1-miR-21-5p-MARCH5 axis positively affects the fission and fusion of mitochondria and cardiomyocyte apoptosis following myocardial I/R. Specifically, lncRNA PVT1 can reduce miR-21-5p expression by directly binding to and being positively regulated by zinc finger protein 36-like 2 (ZFP36L2), which then elevates the E3 ubiquitin ligase MARCH5, exerting a deteriorating effect on myocardial mitochondrial fission and myocardial I/R injury *in vivo* and *in vitro* (44).

In addition to myocardial I/R-induced apoptosis, siRNA and E3 ubiquitin ligase were also associated with ischemia and cardiotoxic-induced apoptosis. Furthermore, Qiu et al. (45) revealed that knockdown of the E3 ubiquitin ligase RNF4 by siRNA could exacerbate cardiac oxidative stress-induced apoptosis and ischemia-induced injury by upregulating the PML nuclear body and promoting p53 activity in mouse models *in vivo*. Moreover, the knockdown of the E3 ubiquitin ligase CHIP by siRNA promoted doxorubicin (DOX)-induced cardiac oxidative stress and injury in the mouse heart *in vivo* (46). Further investigations have illustrated that CHIP can ameliorate cardiac toxicity by facilitating ubiquitin-mediated degradation of SHP-1 and p53 and activating the STAT3 and ERK1/2 pathways.

In addition to ischemia- or I/R-induced cardiomyocyte apoptosis, miRNAs and circRNAs are also involved in the progression of MI through interactions with the UPS. It was demonstrated that miRNA-1 was overexpressed in male mice *in vivo* and *in vitro* during MI (47). Mechanistically, miRNA-1 extensively induces the expression of UPS components, such as 19S, 20S and ubiquitin ligase E3, and accumulates ubiquitin-positive proteins, ultimately exacerbating cardiac apoptosis and remodeling in cardiomyocytes. In contrast, suppression of miR-32-3p enhances coronary atherosclerotic plaque instability, promotes endoplasmic reticulum stress-regulated cardiomyocyte apoptosis, and aggravates acute MI by increasing ubiquitin ligase RNF13 expression in rats *in vivo* (48). Moreover, circNfix was validated as a proapoptotic factor for MI and inhibited cardiomyocyte proliferation and angiogenesis in rats and mice *in vivo* (49). Regarding the regulatory mechanism, circNfix enhances the ubiquitinated degradation of Ybx1 via the E3 ubiquitin ligase Nedd4l, and circNfix also sponges miR-214 and facilitates the expression of Gsk3β, a downstream effector associated with cardiac angiogenesis.

### 3.2 Cardiomyopathy and myocarditis

Cardiomyopathy can be classified as primary (genetic) and secondary, resulting in different phenotypes, including dilated, hypertrophic, diabetes and age-related cardiomyopathy (50, 51). Dilated cardiomyopathy is typically characterized by left ventricle dilation, which reduces systolic function and appears as an HF symptom (32, 52). Diabetic cardiomyopathy represents a metabolic disease associated with independent risk factors such as hyperinsulinemia, hyperglycemia and insulin resistance (53). Moreover, hypertrophic cardiomyopathy is a heterogeneous genetic disease resulting in ventricular hypertrophy, hypercontractility and fibrosis (54), while cardiac hypertrophy is an acquired compensatory reaction to both physiological and pathological overload (55).

It was verified that the miR-199/214 cluster was downregulated during dilated cardiomyopathy and that this downregulation exacerbated the loss of heart mass via the activation of the UPS, including ubiquitin E2-ligases Ube2i and Ube2g1 and E3-ligases MuRF-1 and MAFbx, *in vitro* (56). Notably, miR-199/214 cluster expression was positively regulated by the transcription factor TWIST1, which might be used as a preregulator for miR-199/214/UPS-involved dilated cardiomyopathy. In addition, miR‐195‐5p was speculated to inhibit SGK1 and hERG protein expression by enhancing the activity of the E3 ubiquitin ligase Nedd4‐2 during high-glucose induced cardiomyopathy in rat cardiomyocytes *in vitro* (57).

Moreover, miR-485-5p and lncRNAs (NONRATT054243 and NONRATT057160) have been shown to relieve myocardial apoptosis and cardiac hypertrophy through different types of ubiquitination (58, 59). Specifically, miR-485-5p can suppress mitochondrial fission and phenylephrine (PE)-induced cardiac hypertrophy by decreasing the expression of the SUMO E3 ligase MAPL and thus elevating the level of Mfn2 in an *in vivo* mouse model (58). In addition, the E3 ubiquitin ligase Nrdp1, which positively regulates the two core lncRNAs, NONRATT054243 and NONRATT057160, was also revealed to mitigate Ang II-induced cardiac hypertrophy by reducing downstream PIK3R3 expression and AKT phosphorylation (59). Conversely, UBE3A knockdown by siRNAs exacerbated isoproterenol-induced cardiac hypertrophy by activating the TLR4/MMP-9 pathway (60).

In addition to dilated cardiomyopathy, high glucose-induced cardiomyopathy and cardiac hypertrophy, ncRNA (especially miRNA) and the UPS are associated with septic and viral myocarditis. Remarkably, Gong, Li et al. (61) found that transcription factor sex-determining region Y (SRY)-Box 9 (SOX9) negatively regulated miR-96-5p and was significantly increased in sepsis-induced myocarditis in mice *in vivo*. USP7 deubiquitination modification upregulated SOX9, inhibited miR-96-5p expression and facilitated NLRP3 expression, myocardial pyroptosis and injury exacerbation. Furthermore, overexpression of miR-30a facilitates coxsackievirus B3 (CVB3) replication and induces viral myocarditis by suppressing the expression of the E3 ubiquitin ligase TRIM25, inhibiting RIG-I ubiquitination and thus reducing IFN-β activation (62). Additionally, in CVB3-infected cardiomyocytes *in vivo* and *in vitro*, upregulated miR-21 impairs cell-cell junctions via disruption of desmosome and fascial adhesion by reducing the deubiquitinating enzyme YOD1 and enhancing K48-linked polyubiquitination-mediated desmin degradation and subsequently impairing cell-cell junctions (63).

### 3.3 Heart failure

HF is a clinical syndrome that results from long-term cardiac dysfunction, cardiomyocyte hypertrophy and structural remodeling caused by hypoxia, ischemia, metabolic disorders, etc (2, 32).

The following four ncRNAs, miR-379 and miR-199a, miR-146a and lncDACH1 were shown to be upregulated during HF and to aggravate HF via inhibition of the E3 ubiquitin protein Smurf1, ubiquitin-binding enzymes Ube2i and Ube2g1, and SUMO1 and upregulation of Smurf1 *in vivo* and *in vitro* (39, 64–66). Chen’s groups suggested that miR-379 could facilitate cardiomyocyte apoptosis and possibly cause HF due to deficiency of the antiaging gene Klotho by inhibiting Smurf1 expression. Interestingly, exogenous Smurf1 protein could rescue miR379-induced apoptosis in cardiomyocytes (39). Likewise, miR-199a can be negatively regulated by signal transducer and activator of transcription 3 (STAT3), and its overexpression inhibits the expression of Ube2i and Ube2g1, thereby leading to deterioration of cardiomyocyte ultrastructure and HF (64). Specifically, miR-146a is increased in extracellular vesicles derived from failing hearts. The key regulatory pathway involves extracellular vesicle-associated miR-146a transfer into cardiomyocytes from fibroblasts and suppression of SUMO1 expression, thereby impairing cardiac contractile function and aggravating HF (65). Moreover, lncDACH1 was demonstrated to decrease cell shortening and calcium transients, impair myocardial function and consequently lead to HF by directly promoting Smurf1-induced ubiquitination degradation of sarcoplasmic reticulum calcium ATPase 2a (SERCA2a) in mouse hearts (66).

In contrast, Wang et al. (67) observed that miR-454 was significantly decreased in HF rat models *in vivo* and oxidative stress-damaged cardiomyocyte H9c2 cells *in vitro*. Specifically, miR-454 can suppress cardiomyocyte apoptosis by inhibiting TrkA ubiquitination and degradation and activating the cAMP signaling pathway by negatively targeting the ubiquitin E3 ligase NEDD4-2. Similarly, downregulation of miR-129-5p impairs myocardial structure and function during chronic HF by promoting Smurf1 expression and then accelerating ubiquitination degradation of PTEN in chronic HF rat models (68).

### 3.4 Other cardiac diseases

In addition to MI, cardiomyopathy and HF, several research groups have demonstrated a combined role for ncRNAs and ubiquitination in cardiac diseases such as heart aging, myocardial fibrosis, and calcified aortic valve (AV) disease.

**Figure 4:**
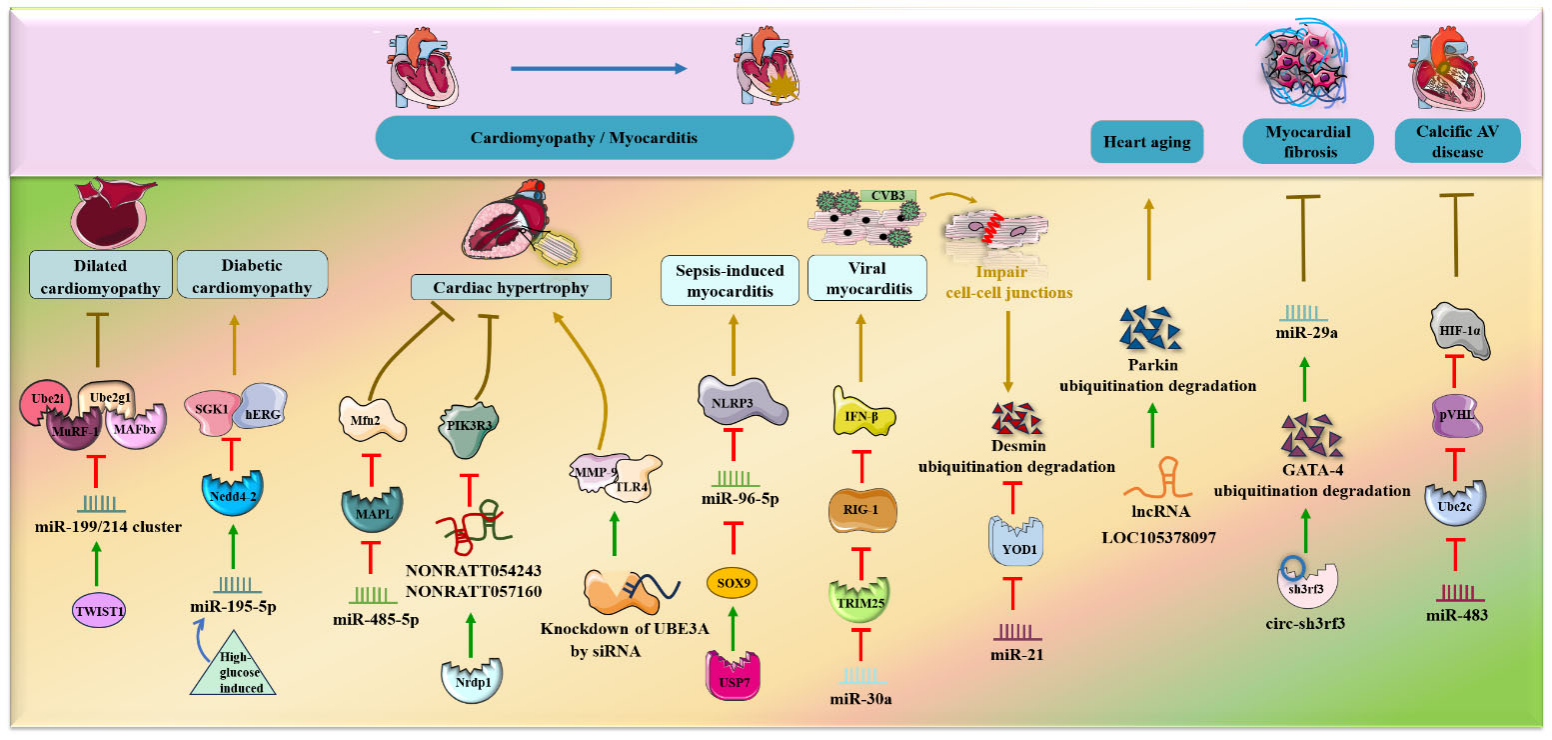
NcRNAs and ubiquitination in other cardiac diseases. The crosstalk of ncRNAs and ubiquitination regulates cardiac diseases, such as hypertrophy, myocarditis, heart failure, heart aging, myocardial fibrosis and calcific AV disease. NcRNAs and ubiquitination are involved in cardiac diseases by regulating target protein expression.

In mouse models *in vivo*, lncRNA LOC105378097 suppressed cardiomyocyte mitophagy and induced heart aging by promoting Parkin ubiquitination and reducing Parkin protein stability (69) (**Figure 4**). CircHIPK3 attenuates heart aging by promoting the binding of E3 ubiquitin ligase β-TrCP to HuR, enhancing ubiquitination and degradation of HuR and ultimately decreasing the activity of p21 in mice *in vivo* and *in vitro* (70). Additionally, it was verified that circ-sh3rf3 inhibited fibroblast-to-myofibroblast differentiation and consequently alleviated myocardial fibrosis by directly enhancing GATA-4 ubiquitination degradation via the E3 ubiquitin–protein ligase sh3rf3 and then abolishing GATA-4 inhibition of miR-29a expression (71). Furthermore, loss of miR-483 contributes to endothelial inflammation and consequently calcific AV disease and exerts deteriorating effects by increasing the expression of Ube2c, enhancing ubiquitination-induced pVHL degradation and activating the HIF-1α pathway (72).

## 4 Crosstalk between ubiquitin ligases and ncRNAs drives vascular disease progression

### 4.1 Atherosclerosis

Atherosclerosis is a chronic inflammatory disease of the blood vessel wall characterized by maladaptive endothelial responses, VSMC structural changes, and macrophage lipid deposition (3, 5, 28). During atherosclerosis development, the major pathological processes are endothelial cell inflammation injury and overgrowth and migration of human VSMCs, in which ubiquitination and ncRNAs are implicated.

**Figure 5:**
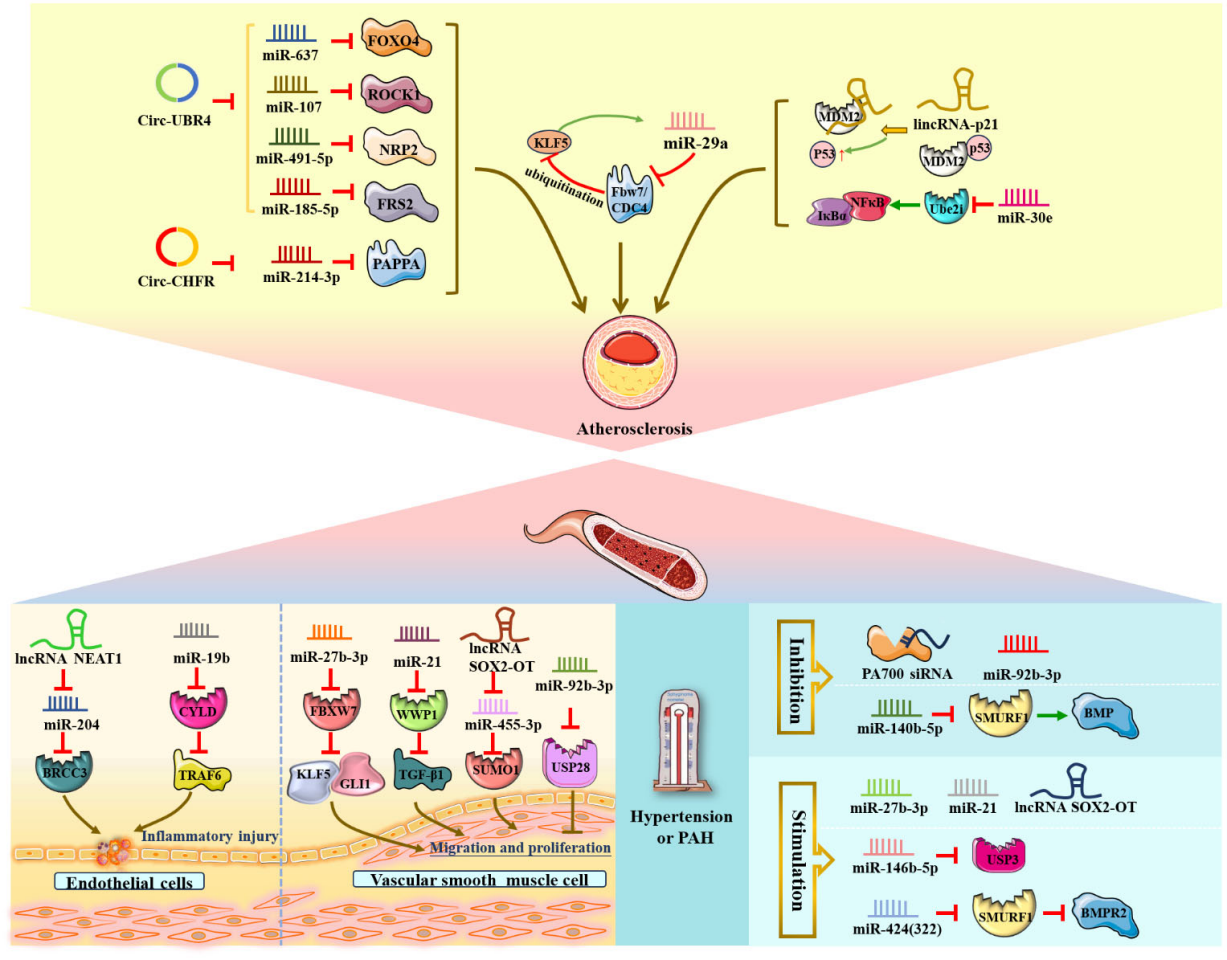
NcRNAs and ubiquitination in vascular diseases. The crosstalk of ncRNAs and ubiquitination regulates vascular diseases, such as endothelial cell inflammatory injury, VSMC proliferation and migration, atherosclerosis, stroke, and hypertension. NcRNAs and ubiquitination are involved in vascular diseases by regulating target protein expression.

The lncRNA NEAT1 and miR-19b mentioned below were shown to aggravate endothelial cell injury and induce an inflammatory response via upregulation of the expression of the deubiquitinating enzyme BRCC3 and inhibition of the expression of the deubiquitinating enzyme CYLD (73, 74) (**Figure 5**). In detail, Yao, Song et al. (73) elucidated that lncRNA NEAT1, as a powerful miR-204 sponge, promoted BRCC3 expression in human umbilical vein endothelial cells (HUVECs) and significantly aggravated H/R-mediated NLRP3 inflammasome-activated endothelial cell injury and pyroptosis. In addition, miR-19b exerted a proinflammatory effect on vascular endothelial cells by reducing the expression of CYLD and further enhancing the accumulation of TRAF6 in spontaneously hypertensive rats *in vivo* (74).

Additionally, circular RNA ubiquitin protein ligase E3 component N-recognin 4 (circ-UBR4), circRNA E3 ubiquitin-protein ligase (circ-CHFR) and circRNA ubiquitin-specific peptidase 36 (circ_USP36) were shown to be upregulated in ox-LDL-induced human VSMCs. Circ-UBR4 sponges miR-637 and reduces the expression of miR-637, thereby promoting FOXO4 production (75, 76). Similarly, circ-UBR4 also promotes human VSMC proliferation by sponging miR-107, miR-491-5p and miR-185-5p and then increasing the expression of ROCK1, NRP2 and FRS2 (77–79). Moreover, circ-CHFR could contribute to human VSMC growth and migration as well as atherosclerosis deterioration via the circ-CHFR/miR-214-3p/PAPPA axis (80). Circ_USP36 exacerbates ox-LDL-induced human endothelial cell dysfunction via circ_USP36/miR-197-3p/ROBO1 axis (81). Notably, Zheng et al. (82) illustrated that miR-29a, Fbw7/CDC4 and KLF5 form a regulatory crosstalk and positive feedback loop that promotes the development of atherosclerosis. Mechanistically, miR-29a could be regulated by KLF5 to reduce Fbw7/CDC4 expression and subsequently enhance the stability of KLF5 by decreasing Fbw7/CDC4-dependent ubiquitination.

Conversely, Zong et al. (83) verified that miR-30e could exert an inhibitory effect on the proliferation and migration of human VSMCs and act as an antiatherosclerotic agent by negatively targeting Ube2i and reducing the IκBα/NFκB signaling pathway in rats *in vivo.* Similarly, lincRNA-p21 functioned as a negative regulator of VSMC proliferation during atherosclerosis and induced VSMC apoptosis by directly binding to the E3 ubiquitin ligase MDM2, decreasing MDM2-p53 interactions and ubiquitination degradation, ultimately promoting p53 activity in mice *in vivo* and *in vitro* (84).

### 4.2 Stroke

Ischemic stroke is a typical clinical condition of cerebrovascular disease due to neuronal damage and destruction of supporting structures caused by cerebrovascular embolism and cerebral infarction (85).

**Figure 6:**
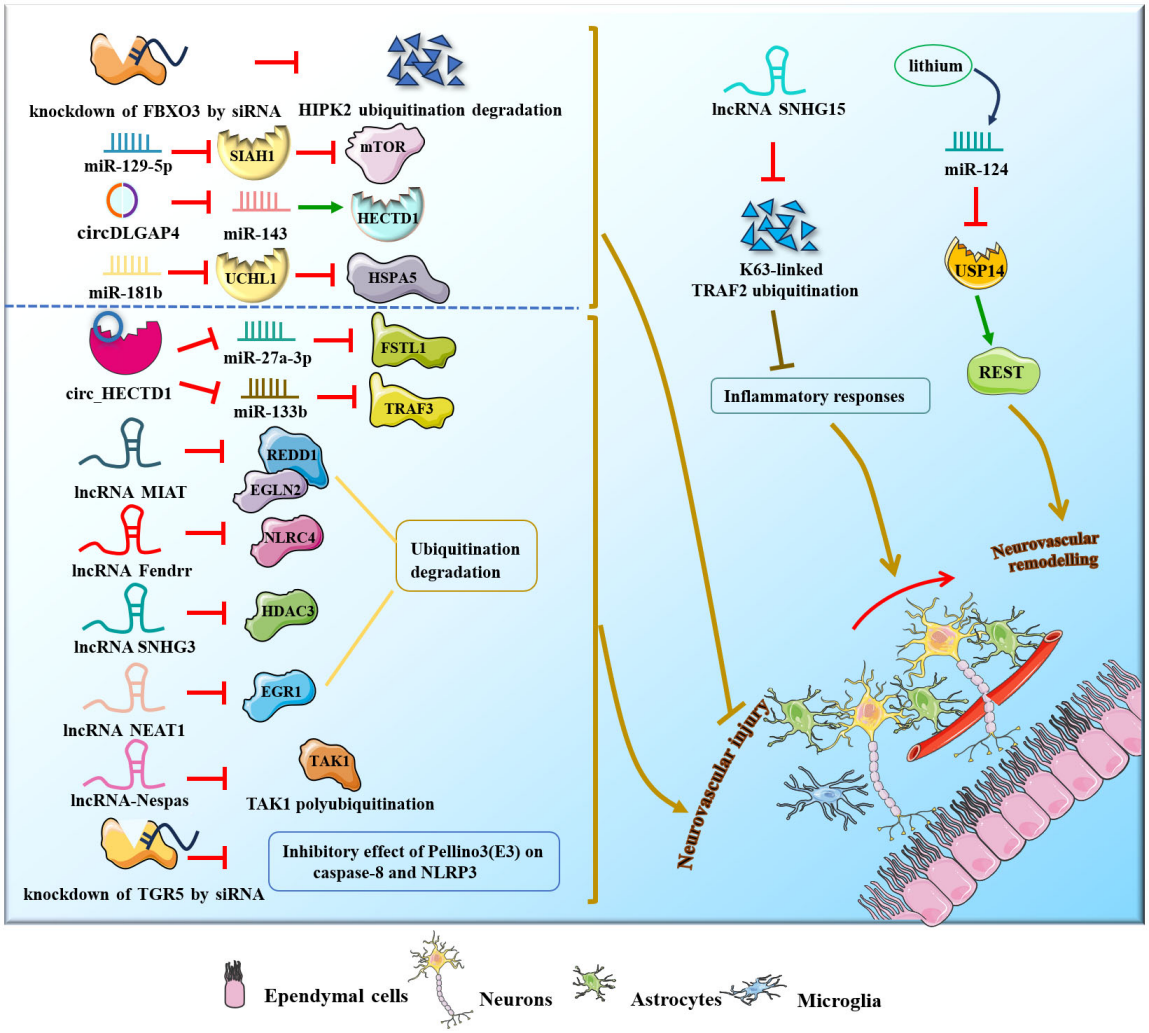
NcRNAs and ubiquitination in stroke.

Several research groups have studied and confirmed the role of miRNAs, circRNAs and lncRNAs in ischemic stroke (**Figure 6**). For example, miR-124 participated in poststroke neurovascular remodeling by directly downregulating the expression of the deubiquitinating enzyme USP14 and reducing REST levels. MiR−129−5p could improve poststroke neurovascular injury via inhibition of the E3 ubiquitin ligase SIAH1 and activation of the mTOR signaling pathway (86–88). Additionally, circDLGAP4 expression was significantly downregulated in both stroke mouse models and plasma from ischemic stroke patients, but overexpression of circDLGAP4 sponged miR-143 and reduced E3 ubiquitin ligase HECTD1 expression, thereby ameliorating poststroke neurovascular injury and reducing infarct size (89). Zhuang, Chen et al. found that circUCK2 suppresses endothelial-mesenchymal transition and protects the blood-brain barrier in ischemic stroke by interacting with FUS to increase the expression of the E3 ubiquitin ligase HECTD1 in mice *in vivo* and *in vitro* (90). Moreover, circSCMH1 promotes translocation of obesity-associated protein into the endothelial cell nucleus via ubiquitination and subsequently facilitates m6A demethylation of Plpp3 mRNA and upregulates Plpp3 expression, thereby enhancing vascular repair after stroke in mice *in vivo* and *in vitro* (91). LncRNA SNHG15 suppressed K63-linked TRAF2 ubiquitination to improve acute ischemic stroke (92). Notably, in mouse ischemic stroke models (middle cerebral artery occlusion) *in vivo*, miR-181b expression is downregulated in response to ischemic exposure, thus ameliorating poststroke neurovascular injury by increasing ubiquitin carboxyl-terminal hydrolase isozyme L1 (UCHL1) and HSPA5 expression (93). However, circRNA HECT domain E3 ubiquitin-protein ligase 1 (circ_HECTD1) could aggravate poststroke neurovascular injury *via* the circ_HECTD1/miR-27a-3p/FSTL1 axis and circ_HECTD1/miR-133b/TRAF3 axis (94, 95). Furthermore, overexpression of lncRNA MIAT and lncRNA-Fendrr reduced ubiquitination and degradation of REDD1 and EGLN2, NLRC4, lncRNA SNHG3 and lncRNA NEAT1 reduced ubiquitination of HDAC3 and EGR1, and lncRNA-Nespas silencing promoted TAK1 polyubiquitination, eventually aggravating cerebral I/R injury and acute ischemic stroke (96–101).

Therapeutically, lithium improved neurovascular remodeling by upregulating miR-124 expression, decreasing REST abundance and inhibiting deubiquitination of peri-infarct brain tissues and proteins on the 4th day post-stroke (102). Inhibition of FBXO3 by siRNA alleviates cerebral I/R injury by suppressing ubiquitination and degradation of HIPK2 via the UPS (103), while TGR5 siRNA reduces the inhibitory effect of the E3 ubiquitin ligase Pellino3 on caspase-8 and NLRP3, thereby exacerbating cerebrovascular stroke (104, 105).

## 5 Crosstalk between ubiquitin ligases and ncRNAs drives in hypertension progression

Presented as an increase in localized pulmonary arterial blood pressure, PAH is characterized by endothelial cell dysfunction, VSMC proliferation, vascular remodeling, and right ventricular overload (106, 107).

VSMCs form the middle layer of arteries and have regulatory functions in the vasculature, such as regulating blood flow, blood pressure and vascular tension (108). Accumulating evidence has illustrated that several miRNAs and lncRNAs are associated with the progression of PAH through the involvement of ubiquitination. Three ncRNAs, miR-27b-3p, miR-21 and lncRNA SOX2-OT, promoted the proliferation of pulmonary artery smooth muscle cells (PASMCs) via downregulation of FBXW7 and WWP1 and upregulation of SUMO1 (109–111). The researchers revealed that overexpression of miR-27b-3p could significantly elevate GLI1 expression through inhibition of ubiquitin ligase FBXW7 expression and accelerating KLF5 accumulation by attenuating its ubiquitination degradation in rats *in vivo* (109). Notably, miR-21 overexpression facilitated chronic hypoxia-induced PASMC proliferation and pulmonary vascular remodeling, and the possible mechanism was that miR-21 negatively regulated the E3 ubiquitin ligase WWP1 and then activated TGF-β1 signaling in mice *in vivo* and *in vitro* (110). Furthermore, Jiang, Hei, et al. (111) identified that increased lncRNA SOX2-OT promoted PASMC growth and migration by negatively reducing downstream miR-455-3p expression and elevating SUMO1 levels. In contrast, miR-92b-3P suppressed the proliferation of PASMCs under hypoxic conditions and improved PAH by inhibiting USP28 expression *in vitro* (112).

In addition, miR-140-5p expression was reduced in rat models *in vivo* and in PAH patients, and knockdown of miR-140-5p could facilitate the expression of the E3 ubiquitin ligase SMURF1 and activate BMP signaling, thereby promoting PASMC proliferation and pulmonary artery endothelial dysfunction and possibly exacerbating PAH (113). Conversely, miR-146-5p facilitated hypoxia-induced proliferation of pulmonary artery endothelial cells and PASMCs by negatively regulating USP3 (114). Specifically, Xu et al. (115) performed a control experiment—knockdown of the 26S proteasome regulatory subunit PA700 by siRNA and modifications of PA700 by tyrosine nitration *in vitro*—and the results indicated that PA700 nitration could promote the combination with the 20S proteasome, activate the 26S proteasome, and reduce GTPCH I and vascular protective enzyme BH4 expression, ultimately damaging endothelial cells in Ang II-induced hypertension. Notably, Baptista et al. discovered that miR-424(322) functioned as a messenger connecting PAH and right ventricle hypertrophy; mechanistically, overexpression of miR-424(322) suppressed SMURF1 expression and activated the BMPR2 pathway in a PAH rat model *in vivo* and in PAH patients, thereby inducing right ventricle overload (116).

## 6 Prognostic and therapeutic targets

### 6.1 Diagnostic indicators of the UPS involving ncRNAs

Increasing evidence has revealed that ncRNAs are in different biofluids, which suggests that ncRNAs have the potential to be used as diagnostic indicators by detecting their dynamic expression (117, 118). Unlike sampling ncRNAs via invasive techniques such as biopsy, it is easy and inexpensive to detect ncRNAs from blood samples (119). However, the current obstacle is the lack of some ncRNAs with sensitivity and specificity (120).

LncRNA LOC105378097, circDLGAP4, miR-140-5p, miR-424(322), miR-146-5p, circ-UBR4 and miR-29a are considered diagnostic indicators of heart aging, ischemic stroke, PAH and atherosclerosis (**Table 1**). LncRNA LOC105378097 is upregulated in the serum of patients with heart aging, which indicates a high diagnostic value (69). Downregulated circDLGAP4 has potential diagnostic significance by sampling plasma from ischemic stroke patients (89). Moreover, reduced expression of miR-140-5p but enhanced miR-424(322) and miR-146-5p expression can be detected in whole blood samples from PAH patients, suggesting their diagnostic significance (113, 114, 116). Additionally, circ-UBR4 and miR-29a, as diagnostic biomarkers, are increased in serum from atherosclerosis patients (75, 77–79, 82).

**Table 1:** ncRNAs as diagnostic indicators for CVDs.

Biofluid	Disease	NcRNA	Dysregulation	Interaction with E3/DUBS	Reaction axis	Ref.
Serum	Heart aging	lncRNA LOC105378097	upregulated	Promote Parkin ubiquitination degradation	lncRNA LOC105378097/Parkin	(69)
Plasma	Ischemic stroke	circDLGAP4	downregulated	Promote HECTD1 expression	circDLGAP4/miR-143/HECTD1	(89)
Whole blood	PAH	miR-140-5p	downregulated	Promote SMURF1 expression	miR-140-5p/SMURF1/BMP	(113)
Plasma	PAH	miR-424(322)	upregulated	Inhibit SMURF1 expression	miR-424(322)/SMURF1	(116)
Serum	PAH	miR-146-5p	upregulated	Inhibit USP3 expression	miR-146-5p/USP3	(114)
Serum	Atherosclerosis	Circ-UBR4	upregulated	Circ-UBR4 negatively regulates miR-637/miR-107/miR-491-5p/miR-185-5p	Circ-UBR4/miR-637/FOXO4; circ-UBR4/miR-107/ROCK1; Circ-UBR4/miR-491-5p/NRP2; circ-UBR4/miR-185-5p/FRS2	(75–79)
Serum	Atherosclerosis	miR-29a	upregulated	Inhibit Fbw7/CDC4-dependent ubiquitination	KLF5/miR-29a/Fbw7/CDC4/KLF5	(82)

Intriguingly, numerous clinical studies have shown that ncRNAs serve as diagnostic markers for CVDs. By using various screening approaches, such as single-cell sequencing and functional high-throughput screening, ncRNA molecules identified in body fluids can be validated in large cohorts of patient samples and used as novel biomarkers for CVDs (121). For example, miR-423-5p and lncRNA LIPCAR might be biomarkers for heart failure in patients’ plasma; miR-1, miR-133, miR-423, miR-208b, miR-499 and lncRNA UCA1, LIPCAR might be used as biomarkers for acute myocardial infarction; miR-155 and miR-17 might serve as biomarkers for coronary artery disease in blood or urine (28, 122, 123). Circ-STAT3 might be a predictor for stroke functional outcomes in 982 patients’ analysis for stroke recovery (124). However, ncRNA-based diagnostic strategies for CVDs are still in the experimental and preclinical stages due to ncRNA variability, analytical and technical factors, relevant isolation methods, cross-platform standardization and accuracy (28), therefore, ncRNA-based diagnostic markers for CVDs may be a supplement and development program for early screening and accurate and comprehensive diagnosis of CVDs in the future.

### 6.2 Therapeutic target of UPS and ncRNAs

Advanced studies have shown that therapeutic strategies based on specific ncRNA modulation are promising candidates to improve CVDs (28, 125, 126). NcRNAs are also considered to be key novel regulators of cardiovascular function and risk factors, potentially serving as small-molecule targets (e.g., miRNAs, lncRNAs, circRNAs and lincRNAs) or gene silencing tools (e.g., siRNAs) for cardiovascular treatment and prognosis assessment (23, 28). Specifically, RNA interference (RNAi) drugs and further novel drugs that mimic or inhibit endogenous ncRNAs may lay the blueprint for the treatment of CVDs at the small molecule level by utilizing molecular interactions, particularly ubiquitination or deubiquitination.

On the first level, ncRNAs are involved in the pathological processes of CVDs and function as potential therapeutic targets by directly regulating E2/E3/deubiquitinating enzyme levels and affecting downstream protein expression (**Table 2**). Various ncRNAs exert cardioprotective effects by inhibiting downstream proteins of ubiquitination degradation (miR322, miR-322/503, miR-378a-3p, miR-454, miR-129-5p) or promoting ubiquitination degradation (lncCIRPIL, LNC01588) (36–38, 41, 43, 67, 68). For example, miR322, miR-322/503, miR-378a-3p, lncCIRPIL, and LNC01588 were shown to exert anti-apoptotic effects to alleviate myocardial I/R progression by inhibiting downstream proteins of FBXW7/Smurf2/TRIM55 ubiquitination or promoting ubiquitination degradation of p53/SEP4 (36–38, 41, 43). MiR322/503 (homolog of human miR-424) reduces myocardial I/R-induced apoptosis and infarct size via the miR-424(322)/503/FBXW7/N1-ICD/HIF-1α and miR-424(322)/503/Smurf2/EZH2 axes (36, 37). Additionally, lncCIRPIL can protect cardiomyocytes from A/R-induced apoptosis by promoting ubiquitination degradation of p53 (41). Moreover, LNC01588 was found to inhibit cardiomyocyte apoptosis and oxidative stress via the LNC01588/HNRNPL/WWP2/SEP4 pathway (43). Furthermore, miR-454 exerts anti-apoptosis effects and improves HF via the miR-454/NEDD4-2/TrkA/cAMP axis (67), while miR-129-5p provides potential therapeutic benefits in CHF via modulation of the miR-129-5p/Smurf1/PTEN axis (68).

**Table 2:** ncRNAs as therapeutic targets for CVDs.

CVD	NcRNA Effects	Experimental model	NcRNA	Interaction with E3/DUBS	Reaction axis	Therapeutical effect	Ref.
Heart disease	Protective effects (anti-apoptosis effects)	Myocardial I/R mice; rat H9c2 cardiomyocytes	miR-424(322)/503 downregulated	Promote FBXW7/Smurf2 expression	miR-322/FBXW7/N1-ICD; miR-322/503/Smurf2/EZH2/Akt/p-GSK3β	MiR-424(322)/503 alleviates myocardial I/R-induced apoptosis and infarct size.	(36, 37)
		Myocardial I/R rat; rat H9c2 cardiomyocytes	miR-378a-3p downregulated	Promote TRIM55 expression	miR-378a-3p/TRIM55/DUSP1/JNK	MiR-378a-3p alleviates myocardial I/R-induced apoptosis and injury.	(38)
		Myocardial I/R mice; mouse cardiomyocytes and human AC16 cells	lncCIRPIL downregulated	Inhibit COP1, CHIP and MDM2 expression	lncCIRPIL/COP1, CHIP, MDM2/p53; p53/lncCIRPIL	LncCIRPIL alleviates myocardial A/R-induced apoptosis and injury.	(41)
		Myocardial I/R rat; neonatal rat ventricle myocytes and H9c2 cells	lncRNA Fendrr downregulated	Inhibit the binding of COP1 and p53	lncRNA Fendrr/COP1/p53	LncRNA Fendrr alleviates myocardial H/R-induced apoptosis.	(42)
		Myocardial I/R mice; human AC16 cells	LNC01588 downregulated	Promote WWP2 expression	LNC01588/HNRNPL/WWP2/SEP4; WWP2/LNC01588	LNC01588 alleviates myocardial oxidative stress and myocardial A/R injury.	(43)
	Worsening effects	Myocardial I/R mice model; mouse cardiomyocytes	lncRNA PVT1 upregulated; miR-21-5p downregulated	Promote MARCH5 expression	ZFP36L2/lncRNA PVT1/miR-21-5p/MARCH5	Inhibition of lncRNA PVT1 attenuates the fission and fusion of mitochondria and myocardial I/R injury.	(44)
	pro-apoptotic, pro-fibrotic, and pro-remodeling effects	MI mice	miRNA-1 upregulated	Promote UPS components (19S, 20S and ubiquitin ligase E3) expression	miRNA-1/UPS (19S, 20S and ubiquitin ligase E3)	Inhibition of miRNA-1 attenuates cardiomyocyte apoptosis and cardiac remodeling and improve MI.	(47)
	pro-apoptotic and antiproliferative and antiangiogenic effects	MI mice and rat; neonatal cardiomyocytes	circNfix upregulated	Significantly affect the interaction between Ybx1 and Nedd4l	SE/circNfix/Nedd4l/Ybx1, circNfix/miR-214/Gsk3β	Inhibition of circNfix attenuates cardiomyocyte apoptosis, facilitates cardiomyocyte proliferation and angiogenesis and induces cardiac regeneration	(49)
	pro-apoptotic effects (RNF4 siRNA)	MI mice; neonatal cardiomyocytes	siRNA	Knockdown RNF4 by siRNA	siRNA/RNF4/PML/p53	RNF4 alleviates cardiac oxidative stress-induced apoptosis and ischemia-induced injury	(45)
	Protective effects(anti-apoptosis)	Arterial plaque rats; embolization rats	miR-32-3p upregulated	Inhibit RNF13 expression	miR-32-3p/RNF13	MiR-32-3p alleviates endoplasmic reticulum stress-regulated cardiomyocyte apoptosis, promotes atherosclerotic plaque stability and improves acute MI.	(48)
	Protective effects	Neonatal rat cardiomyocytes and tissue from human left ventricle	miR-199/214 downregulated	Promote UPS (ubiquitin E2- and E3-ligase) expression	TWIST1/miR-199/214/UPS	miR-199/214 improves heart mass and dilated cardiomyopathy.	(56)
	Worsening effects (CHIP siRNA)	mouse hearts and newborn cardiomyocytes	siRNA	Knockdown CHIP by siRNA (DOX downregulate the CHIP expression)	CHIP/SHP-1 and p53/STAT3and ERK1/2	CHIP alleviates DOX- induced cardiac toxicity and cardiac injury.	(46)
	Worsening effects	high-glucose-induced diabetic cardiomyocytes	miR-195-5p upregulated	Promote Nedd4-2 expression (inhibition of SGK1activates Nedd4-2)	miR-195-5p/SGK1/Nedd4-2/hERG	Inhibition of miR-195-5p attenuates high-glucose induced cardiomyopathy.	(57)
	Protective effects	PE-induced cardiomyocyte hypertrophy mice; neonatal rat cardiomyocytes	miR-485-5p downregulated	Promote MAPL expression	miR-485-5p/MAPL/Mfn2	MiR-485-5p alleviates mitochondrial fission and cardiomyocyte hypertrophy.	(58)
	Protective effects (Nrdp1)	Ang-II- treated neonatal rat cardiomyocytes	two core lncRNAs (NONRATT054243 and NONRATT057160) downregulated	Nrdp1 positively regulates two core lncRNAs	Nrdp1/NONRATT054243 and NONRATT057160/PIK3R3/AKT	Nrdp1 alleviates Ang II-induced cardiomyocyte hypertrophy.	(59)
	Worsening effects (UBE3A siRNA)	Isoproterenol-treated human AC16 cells	siRNA	Knockdown UBE3A by siRNA	UBE3A/TLR4/MMP-9	UBE3A alleviates isoproterenol-induced cardiomyocyte hypertrophy	(60)
	pro-apoptotic effects	Klotho- deficient mice; H9c2 cells	miR-379 upregulated	Inhibition smurf1 expression	miR-379/Smurf1; Smurf1/miR-379	Inhibition of miR-379 attenuates cardiomyocyte apoptosis and improves HF	(39)
	Worsening effects	STAT3- deficient mice; neonatal rat cardiomyocytes	miR-199a upregulated	Inhibit Ube2i and Ube2g1(UPS)	STAT3/miR-199a/Ube2i and Ube2g1	Inhibition of miR-199a attenuates cardiomyocyte ultrastructure and improves HF.	(64)
		extracellular vesicle-treated failing cardiomyocytes	MiR-146a upregulated	Inhibit SUMO1 expression	MiR-146a/SUMO1	Inhibition of miR-146a improves cardiac contractile function and HF.	(65)
		LncDACH1 overexpressing mice; human cardiac samples and cardiomyocytes	LncDACH1 upregulated	Promote SERCA2a ubiquitination degradation	LncDACH1/SERCA2a	Inhibition of lncDACH1 improves myocardial function and alleviates HF.	(66)
	Protective effects ( anti-apoptosis effects)	HF rat models; oxidative stress -induced cardiomyocyte H9c2 cells	miR-454 downregulated	Promote NEDD4- 2 expression	miR-454/NEDD4- 2/TrkA/cAMP	MiR-454 alleviates cardiomyocyte apoptosis and improve HF.	(67)
	Protective effects	chronic HF rats; primary rat cardiomyocytes	miR-129-5p downregulated	Promote Smurf1 expression	miR-129-5p/Smurf1/PTEN	MiR-129-5p improves myocardial structure and chronic HF.	(68)
	Protective effects	isoproterenol-treated rat cardiac fibroblasts and cardiomyocytes	Circ-sh3rf3 downregulated	Inhibit GATA-4 ubiquitination degradation via sh3rf3	Circ-sh3rf3/sh3rf3/GATA-4/miR-29a	Circ-sh3rf3 inhibits the fibroblast-to-myofibroblast differentiation and improves myocardial fibrosis	(71)
	Anti-inflammatory effects	HAVECs and pig isolated hearts	miR-483 downregulated	Promote UBE2C expression	miR-483/UBE2C/pVHL/HIF-1α	MiR-483 alleviates endothelial inflammation and improves calcific aortic valve (AV) disease.	(72)
	Worsening effects (SOX9)	A murine model of sepsis; HL-1 mouse cardiomyocytes	miR-96-5p downregulated	Deubiquitinating enzyme USP7 upregulated (USP7 modifies SOX9)	USP7/SOX9/miR-96-5p/NLRP3	Inhibition of SOX9 attenuates myocardial pyroptosis and injury	(61)
	Worsening effects	HeLa cells, 293T cells and mice primary cardiomyocytes and CVB3	miR-30a upregulated	Inhibit TRIM25 expression	miR-30a/TRIM25/IFN-b	Inhibition of miR-30a attenuates CVB3 replication and improves viral myocarditis.	(62)
		Cvb3-infected mice; immortalized human cardiomyocytes	miR-21 upregulated	Inhibit deubiquitinating enzyme YOD1 expression	miR-21/YOD1/desmin	Inhibition of miR-21 attenuates Cvb3-infected injury and protects cell-cell junctions.	(63)
vascular disease	Worsening effects	H/R HUVECs	lncRNA NEAT1 upregulated	Promote deubiquitinating enzyme BRCC3 expression	NEAT1/miR-204/BRCC3	Inhibition of NEAT1 attenuates H/R-mediated endothelial cell injury and cell pyroptosis	(73)
	pro-inflammatory effects	spontaneously hypertensive rats	miR-19b upregulated	Inhibit deubiquitinating enzyme CYLD expression	miR-19b/CYLD/TRAF6	Inhibition of miR-19b attenuates inflammatory responces.	(74)
	Worsening effects (PA700 nitration)	Ang II–Induced hypertension rats; HUVECs	siRNA	Knockdown PA700 by siRNA inhibits the combination with 20S and decreases 26S activation.	PA700/20S proteasome/26S proteasome/GTPCH I and BH4	PA700 siRNA attenuates endothelial cell injury in Ang II-induced hypertension.	(115)
	Proliferative effects on PASMCs	PAH rats; PASMCs	miR-27b-3p upregulated	Inhibit FBXW7 expression	miR-27b-3p/FBXW7/KLF5/GLI1	Inhibition of miR-27b-3p attenuates PASMC proliferation and improves PAH.	(109)
		Mice of hypoxic exposure; human primary PASMCs	miR-21 upregulated	Inhibit WWP1 expression	miR-21/WWP1/TGF-β1	Inhibition of miR-21 attenuates PASMC proliferation and pulmonary vascular remodelling.	(110)
		hypoxic-induced human PASMCs; clinical PAH patients	lncRNA SOX2-OT upregulated	Promote SUMO1 expression	lncRNA SOX2-OT/miR-455-3p/SUMO1	Inhibition of lncRNA SOX2-OT attenuates PASMC proliferation and migration.	(111)
	Inhibitory effects on PASMC proliferation	hypoxia-induced rat PASMCs	miR-92b-3P downregulated	Promote deubiquitinating enzymes USP28 expression	miR-92b-3P/USP28	MiR-92b-3P attenuates PASMC proliferation under hypoxia conditions and improves PAH.	(112)
	Proliferative effects on HVSMCs	HA-VSMCs treated with ox-LDL	miR-214-3p downregulated	Circ-CHFR upregulated (circ-CHFR negatively regulates miR-214-3p)	circ-CHFR/miR-214-3p/PAPPA	Inhibition of circ_ CHFR attenuates HVSMC proliferation and migration.	(80)
	anti-atherosclerotic	carotid artery balloon injury rats; rat VSMCs	miR-30e downregulated	Promoting Ube2i expression	miR-30e/Ube2i/IκBα/NFκB	miR-30e alleviates VSMC proliferation and migration and promotes HVSMC apoptosis	(83)
		injury-induced mice of carotid artery neointimal hyperplasia; HVSMCs	lincRNA-p21 downregulated	Promote lincRNA-p21-MDM2 interaction	lincRNA-p21/MDM2/p53	lincRNA-p21 alleviates HVSMC proliferation and migration and promotes HVSMC apoptosis	(84)
	neuroprotective effects	middle cerebral artery occlusion (MCAO) mice; oxygen glucose deprivation (OGD)-induced neuronal cells	miR-124 downregulated	Promote deubiquitinating enzyme Usp14 expression	miR-124/Usp14/REST	MiR-124 promotes post-stroke neuroprotection and neurovascular remodeling	(86, 87)
			miR-129-5p downregulated	Promote SIAH1 expression	miR-129-5p/SIAH1/mTOR	MiR-129-5p alleviates neuronal cell apoptosis and improves ischemic brain injury	(88)
	inhibitory effects on neuroprotection	MCAO mice; OGD-induced N2A neuroblastoma cells	miR-181b downregulated (in response to ischemic exposure)	Promote UCHL1 expression	miR-181b/UCHL1 and HSPA5	Inhibition of miR-181b protects neurons and improves ischemic stroke.	(93)
	inhibitory effects on neuroprotection (circ_HECTD1)	MCAO mice; OGD-induced neuronal cells	miR-133b/miR-27a-3p downregulated	circ_HECTD1 upregulated (circ_HECTD1 negatively regulates miR-133b/miR-27a-3p)	circ-HECTD1/miR-133b/TRAF3; circ_HECTD1/miR-27a-3p/FSTL1	Inhibition of circ-HECTD1 attenuates neuron apoptosis, promotes cell proliferation and improves ischemic stroke.	(94, 95)
	inhibitory effects on neuroprotection	MCAO rats; OGD-induced neuronal cells	lncRNA MIAT upregulated	Inhibit REDD1/EGLN2 ubiquitination degradation	lncRNA MIAT/REDD1; lncRNA MIAT/EGLN2	Inhibition of LncRNA MIAT attenuates neuron apoptosis and autophagy and improves ischemic stroke	(96, 97)
	Worsening effects	MCAO mice; OGD-induced cells	lncRNA SNHG3 upregulated	Inhibit HDAC3 ubiquitination degradation	LncRNA SNHG3/HDAC3	Inhibition of lncRNA SNHG3 attenuates cerebral I/R injury and inhibits inflammatory factors secretion.	(98)
			lncRNA NEAT1 upregulated	Inhibit EGR1 ubiquitination degradation	lncRNA NEAT1/EGR1/RBM25	Inhibition of lncRNA NEAT1 attenuates neuronal injury, decreases infarct size and improves ischemic stroke.	(99)
		diabetic cerebral I/R mice; mouse microglia cell line BV-2 cells	lncRNA-Fendrr upregulated	Inhibit HERC2 expression	lncRNA-Fendrr/HERC2/NLRC4	Inhibition of lncRNA-Fendrr attenuates neurological deficits and inhibits microglia pyroptosis.	(100)
	Anti- inflammatory, anti-apoptosis effects	cerebral I/R mice; microglial cells from mouse brain	lncRNA-Nespas downregulated	Promote TAK1 polyubiquitination	lncRNA-Nespas/TAK1/NF-κB	lncRNA-Nespas alleviates cerebral I/R-Induced neuroinflammation, microglial cell death and ischemic stroke.	(101)
	Anti- inflammatory effects	MCAO mice and human blood samples; human monocytes/macrophages	SNHG15 upregulated (post-stroke)	Inhibit TRAF2 ubiquitination degradation	SNHG15/TRAF2	SNHG15 alleviates inflammatory response after acute ischemic stroke	(92)
	neuroprotective effects (Lithium)	ischemic stroke mice;OGD-induced neurons treated with Lithium	miR-124 upregulated	Inhibit deubiquitination and proteins	Lithium/miR-124/REST	Lithium protects neuron survival.	(102)
	anti-inflammatory effects (FBXO3 siRNA)	MCAO rats; OGD-induced neuronal cell line HT22 cells	siRNA	Knockdown FBXO3 by siRNA	FBXO3/HIPK2	Inhibition of FBXO3 by siRNA attenuates neuroinflammation and improves cerebral I/R injury.	(103)

In addition, several ncRNAs are directly involved in improving vascular diseases by inhibiting the ubiquitination of downstream proteins (miR-30e, circDLGAP4, lncRNA-Nespas, SNHG15, miR-92b-3p) or promoting binding to the ubiquitin ligase(lincRNA-p21) (83, 84, 89, 92, 101, 112). MiR-30e and lincRNA-p21 act as potential targets for the treatment of atherosclerosis by inhibiting human VSMC proliferation and migration, respectively, via the miR-30e/Ube2i/IκBα/NFκB and lincRNA-p21/MDM2/p53 axes (83, 84). Notably, circDLGAP4, lncRNA-Nespas, and SNHG15 can exert neuroprotective effects to alleviate ischemic stroke by decreasing the expression of the E3 ubiquitin ligase HECTD1 or inhibiting the ubiquitination of TAK1/TRAF2 (89, 92, 101). Interestingly, circDLGAP4 serves as a therapeutic target for ischemic stroke and a biomarker of disease activity via regulation of the circDLGAP4/miR-143/HECTD1 axis and plasma detection (89). LncRNA-Nespas can improve the prognosis of ischemic stroke via the lncRNA-Nespas/TAK1/NF-κB pathway (101). Furthermore, miR-92b-3P can be a potential therapeutic benefit for PAH treatment by suppressing the expression of the deubiquitinating enzyme USP28 (112).

Unlike the ncRNAs mentioned above, which directly exert protective effects on hearts and blood vessels, many ncRNAs are suppressed to improve CVDs by regulating UPS components. For instance, knockdown of ncRNAs was considered a promising therapeutic strategy for the treatment of heart diseases by suppressing ubiquitination degradation of proteins (miRNA-1, circNfix, miR‐195‐5p, lncRNA LOC105378097, lncDACH1) or promoting ubiquitination enzyme expression (miR-30a, miR-21) (47, 49, 57, 62, 63, 66, 69). Silencing of miRNA-1 and circNfix plays important roles in inhibiting cardiomyocyte apoptosis and alleviating the MI process by inhibiting UPS components/Nedd4l (47, 49). Interestingly, a reduction in SE-regulated circNfix has proliferative and proangiogenic effects and improves MI prognosis via the SE/circNfix/Nedd4l/Ybx1 signaling pathway and circNfix/miR-214/Gsk3β axis (49). In addition, knockdown of miR‐195‐5p and lncDACH1 can prevent and treat diabetic cardiomyopathy and HF, respectively, by regulating the miR‐195‐5p/SGK1/Nedd4‐2 axis or enhancing SERCA2 ubiquitination and degradation (57, 66). Silencing of lncRNA LOC105378097 has protective effects against heart aging by facilitating Parkin ubiquitination.

Moreover, inhibition of miR-30a and miR-21 can suppress CVB3 replication and reduce CVB3 infection-induced injury by promoting the expression of the E3 ubiquitin ligase TRIM25/deubiquitinating enzyme YOD1 (62, 63).

For the treatment of vascular diseases, silencing of ncRNAs provides potential therapeutic strategies by inducing ubiquitination degradation (miR-29a, miR-181b, lncRNA-Fendrr, lncRNA MIAT, lncRNA SNHG, miR-21, miR-424(322)) or inhibiting the deubiquitinating enzyme BRCC3/SUMO1 (lncRNA NEAT1, lncRNA SOX2-OT) (73, 82, 93, 96, 98, 100, 110, 111, 116). First, knocking down lncRNA NEAT1 can exert anti-inflammatory effects and protect endothelial cells by regulating the miR−204/BRCC3 axis (73). To attenuate atherosclerosis, inhibition of miR-29a is an effective therapeutic approach by modulating the KLF5/miR-29a/Fbw7/CDC4/KLF5 positive feedback loop (82). Additionally, inhibition of miR-181b, lncRNA-Fendrr, lncRNA MIAT and lncRNA SNHG3 was determined to play neuroprotective roles in treating cerebral I/R injury and ischemic stroke by promoting UCHL1/HERC2 expression or accelerating REDD1/HDAC3 ubiquitination degradation (93, 96, 98, 100). Specifically, knockdown of miR-181b and lncRNA-Fendrr can enhance ischemic injury-induced neuroprotection by the miR-181b/UCHL1/HSPA5 axis and lncRNA-Fendrr/HERC2/NLRC4 axis (93, 100). Furthermore, suppression of miR-21, miR-424(322) and lncRNA SOX2-OT offers potential therapeutic benefits in PAH by upregulating WWP1/SMURF1 or downregulating SUMO1 (110, 111, 116). Decreased miR-21 and lncRNA SOX2-OT prevent PASMC proliferation and pulmonary vascular remodeling, respectively, by the miR-21/WWP1/TGF-β1 signaling pathway and SOX2-OT/miR-455-3p/SUMO1 axis, and lncRNA SOX2-OT also serves as a novel biomarker for the diagnosis of PAH (110, 111). Interestingly, miR-424(322) detection in plasma can function as a diagnostic biomarker for PAH and improve prognosis via the miR-424(322)/SMURF1/BMPR2 pathway (116).

This is particularly noteworthy that circRNA, as an emerging ncRNA, has received much attention in recent years. The mechanism is that circRNAs are involved in CVD progression by sponging miRNAs and regulating downstream proteins (circRNA/miRNA/ubiquitin ligase or deubiquitinating enzymes/protein axis) via ubiquitination, for example, circDLGAP4/miR-143/HECTD1 axis improved stroke (89), circ-UBR4/miR-637/FOXO4, circ-UBR4/miR-107/ROCK1, circ-UBR4/miR-491-5p/NRP2, circ-UBR4/miR-185-5p/FRS2, and circ-CHFR/miR-214-3p/PAPPA axes as well as inhibition of circ_USP36/miR-197-3p/ROBO1 axis could ameliorate atherosclerosis (75–81), circ-HECTD1/miR-133b/TRAF3 and circ_HECTD1/miR-27a-3p/FSTL1 axes as well as inhibition of circNfix/Nedd4l/Ybx1 and circNfix/miR-214/Gsk3β axes could reduce myocardial infarction and CVD (49, 94, 95), circ-sh3rf3/sh3rf3/GATA-4/miR-29a alleviated cardiac fibrosis (71).

On the second level, some ubiquitin ligase mimics or inhibitors serve as therapeutic targets, providing new perspectives for the treatment of CVDs. Nrdp1, TRIM55 inhibitors and Circ_HECTD1 inhibitors can improve CVDs by being negatively regulated by miR-378a-3p, positively regulating NONRATT054243 and NONRATT057160 or negatively regulating miR-27a-3p and miR-133b (38, 59, 94, 95). For instance, Nrdp1 may have potential therapeutic effects on cardiomyocyte hypertrophy via the Nrdp1/NONRATT054243 and NONRATT057160 axes (59). Conversely, TRIM55 silencing inhibits cardiomyocyte apoptosis via the downstream DUSP1/JNK pathway for the treatment of myocardial I/R injury (38). Circ_HECTD1 deficiency offers a novel preclinical basis for cerebral ischemia injury and cerebral infarction via the circ_HECTD1/miR-27a-3p/FSTL1 axis and circ_HECTD1/miR-133b/TRAF3 axis (94, 95). In addition, many studies determined the mechanism and manifestation of CVD after ubiquitin ligases RNF4, CHIP, UBE3A and FBXO3 knockdown by siRNA, suggesting that these ubiquitin ligases can function as targets for CVD treatment. RNF4 suppresses ischemia-induced cardiomyocyte apoptosis via the PML/p53 axis (45). Similarly, CHIP provides a therapeutic method for DOX-induced HF via CHIP/SHP-1 and p53/STAT3 and ERK1/2 (46). UBE3A is vital for alleviating cardiac hypertrophy via the UBE3A/TLR4/MMP-9 pathway (60). In contrast, inhibition of FBXO3 exerts anti-inflammatory effects and relieves ischemic stroke by suppressing of HIPK2 ubiquitination and degradation (103).

On the third level, therapeutic targets are not only ncRNAs or UPS, and there are several ncRNAs, UPS components or downstream proteins that work together as targets for the treatment of CVDs. The details are as follows: miR379 and Smurf1, miR-199a and specific UPS components, miR-146a and SUMOylation were shown to participate in the treatment of HF (39, 64, 65); HIF-1α pathway inhibitors and miR-483 mimics can be potential therapeutic agents for calcific AV disease (72); miR-140-5p and SMURF1 are emerging as crucial regulators and therapeutic targets for PAH (113). Additionally, miR-129-5p and SIAH1 can be therapeutic and preventive targets in cerebral ischemic injury via the miR−129−5p/SIAH1/mTOR signalling pathway (88). Similarly, miR-124 and REST provide therapeutic benefits in protecting against stroke via miR-124/USP14/REST, while other studies have demonstrated that lithium or M2 microglia-derived exosomes can regulate miR-124 and REST to play a neuroprotective role (86, 87, 102). Cardiomyocyte hypertrophy and myocardial fibrosis can be treated via the regulation of the miR-485-5p/MAPL/Mfn2 axis and Circ-sh3rf3/GATA-4/miR-29a cascade (58, 71). Moreover, five notable crosstalk pathways, circ-UBR4/miR-637/FOXO4, circ-UBR4/miR-107/ROCK1, circ-UBR4/miR-491-5p/NRP2, circ-UBR4/miR-185-5p/FRS2 and circ-CHFR/miR-214-3p/PAPPA axis, regulate VSMC migration and growth to improve atherosclerosis (75, 77–80). Furthermore, regulating the MIAT/EGLN2 axis and NEAT1/EGR1/RBM25 axis provides therapeutic strategies for ischemic stroke therapy (97, 99), and ETAR/miR-27b-3p/FBXW7/KLF5/GLI1 axis and miRNA-146-5p/USP3 axis may act as potential targets for PAH prevention and treatment (109, 114).

On the fourth level, targeting upstream proteins, which are regulators of crosstalk between ncRNA and the UPS, represents a promising and novel approach to CVD treatment. Specifically, ZFP36L2 silencing improves myocardial I/R injury via regulation of the lncRNA PVT1/miR-21-5p/MARCH5 axis (44). In the treatment of dilated cardiomyopathy, TWIST1 acts as an advantageous target to improve heart quality by increasing miR-199/214 cluster expression and decreasing UPS activity (56). Interestingly, SOX9 is regulated by USP7 and has therapeutic value for sepsis-induced myocardial damage by regulating the downstream miR-96-5p/NLRP3 pathway (61). For vascular diseases, soybean-derived vasoactive peptide protects vascular endothelial cells from inflammatory damage via manipulation of the miR-19b/CYLD/TRAF6 axis (74). TGR5 has an anti-neuroinflammatory role in the clinical treatment of poststroke via the TGR5/Pellino3/caspase-8 and NLRP3 axes (104). Furthermore, PA700 inhibitors are effective for Ang II-induced hypertension therapy via regulation of 20S,26S proteasomes/GTPCH I and BH4 (115).

Nucleic acid drugs based on various disease-associated ncRNAs provide a new perspective in disease therapy. NcRNA-based drugs are designed to exert therapeutic effects by interacting with proteins or nucleic acids (127). However, this type of drug has two notable limitations. First, RNA molecules may evoke the host’s immune system and result in adverse immune responses. Second, because of the general nature of their targets, ncRNA-based drugs might not target specific sites (125). Therefore, current studies have proposed that ncRNAs combined with biocompatible nanoparticles (NPs) can offer better therapeutic properties. Using NPs as delivery vectors can reduce drug toxicity, facilitate targeted drug release, and increase drug bioavailability, which provides an optimized therapeutic strategy for the cardiovascular field (125, 128–130). In addition, with the production of proteasome inhibitors, the UPS offers a new therapeutic frontier. Drugs targeting ubiquitin ligases were found to have few side effects and significant treatment outcomes (15).

Notably, there have been various clinical trials using ncRNA or ubiquitin ligases for the treatment of CVDs. For instance, MiR-132 inhibitor CDR132L as a novel antisense therapy drug was demonstrated to alleviate heart failure and cardiac remodelling in first clinical trial (131). In the PREDIMED randomized controlled trial, a high-unsaturated fat medicine diet intervention, decreasing triglycerides, possibly reducing stroke risk in miRNA-410 target rs13702 C allele carriers (132). Moreover, the randomized controlled study found that the concentrations of HDL-carried miR-223 and miR-135a were elevated in healthy men following intake of trans fatty acids, preventing worsening of cardiovascular risk (133). Furthermore, siRNA therapy, Olpasiran, was applied to decrease lipoprotein(a) concentrations, thereby effectively ameliorating atherosclerotic CVDs (134). Similarly, Nissen, Wolski et al. revealed that plasma lipoprotein(a) concentrations are dose-related reduced in the siRNA SLN360 single ascending dose study (135). In addition, the ubiquitin E3 ligase NEDD4 rs4149601 can serve as a predictor of adverse cardiovascular consequences in whites without hydrochlorothiazide intervention, and NEDD4 rs292449, rs75982813 and rs4149601 can act as a predictor of blood pressure response after hydrochlorothiazide administration in Pharmacogenomic Evaluation of Antihypertensive Responses (PEAR) clinical trial (136).

In conclusion, known clinical trials using ncRNAs or ubiquitin ligases for the treatment of CVDs may provide a basis for emerging relevant laboratory studies of crosstalk between ncRNAs and ubiquitin ligases for CVD treatment, moving towards further clinical trials. For example, NEDD4 was found to predict adverse cardiovascular outcomes in PEAR clinical trial (136), and miR-454/NEDD4-2/TrkA/cAMP axis was demonstrated to exert anti-apoptosis effects and improves HF in rats, thus potentially laying the foundation for clinical trials.

## 7 Conclusion and future perspectives

Undoubtedly, CVDs constitute enormous health and socioeconomic burdens worldwide. The UPS is involved in the posttranscriptional modification of proteins. NcRNAs are unique RNA transcripts and play crucial roles in cellular processes and the development of various diseases, including CVDs. In this review, we creatively focused on the role of crosstalk between ncRNAs and the UPS in CVD processes. The main mechanism is that ncRNAs regulate E3 ubiquitin ligase levels and ubiquitination degradation of targeted proteins, and vice versa. A small number of E3 ubiquitin ligases can also regulate ncRNA expression. The crosstalk between ncRNAs and the UPS can affect the development of CVDs, such as myocardial I/R injury, MI, cardiomyopathy, HF, myocarditis, atherosclerosis, HT, and ischemic stroke.

A better knowledge of the role of ncRNAs and the UPS can finally provide promising therapeutic strategies for the treatment of CVDs. (1) Targeting ncRNAs can regulate E3/E2/deubiquitinating enzyme expression and subsequently promote or inhibit ubiquitination degradation of targeted proteins. (2) E3 ubiquitin ligases can act as therapeutic targets by regulating ncRNA expression or directly promoting ubiquitination and degradation of downstream proteins. (3) NcRNAs, UPS components and target proteins can serve as cotherapeutic targets. (4) Targeting upstream regulatory proteins or directly knocking down of E3 ubiquitin ligases by siRNA can have a therapeutic impact on CVD. Specifically, the levels of miR-424(322), miR-140-5p, circDLGAP4, and lncRNA LOC105378097, as well as SMURF1, HECTD1, and Parkin ubiquitination can be measured in clinical PAH, stroke, and cardiac aging patient blood, suggesting their diagnostic value.

In addition to the UPS, ncRNAs interact with other protein degeneration pathways involved in CVD development, such as calpains and autophagy. MiR-124-3 reduces cardiomyocyte viability by increasing the expression of the target calpain1, enhancing caspase-3 activity and down-regulating Bcl-2 (137). MiR-137 suppresses the proliferation of hypoxia-induced PASMC and improved PAH by upregulating calpain-2 expression (138). Moreover, Gao, Chen et al. (139) found that many miRNAs promote cardiomyocyte autophagy and improve cardiac function by upregulating ATGs (LC3 and beclin-1) and generating autophagosomes. Hsa_circ_0030042 sponges target eIF4A3 and regulates abnormal autophagy to enhance atherosclerotic plaque stability (140).

Despite growing evidence from experimental studies demonstrating the potential mechanism and significant implication for ncRNAs and the UPS in CVDs, there are still many limitations. First, the specific mechanism is still incomplete, and there are few evidence-based epidemiologic and clinical studies. Second, individual ncRNA or the UPS has a large number of regulatory roles in CVDs, but some of them remain unclear. Third, compared to the crosstalk between miRNAs and the UPS, knowledge on the relationship between lncRNAs/circRNAs/siRNAs and the UPS is far scarcer. Fourth, it is also unknown whether the mechanism of the interaction between ncRNAs, the UPS and CVDs is indeed widely effective in cardiomyocytes. Fifth, further studies are needed to examine the effects of genetic variability among individuals (e.g. DNA and RNA methylation, ATP-dependent chromatin remodelling, and post-translational histone modifications) on the relationship between ncRNAs, ubiquitin ligases, and CVD progression (141). Sixth, the role of other protein degradation pathways regulated by ncRNAs (e.g. calpain and autophagy) in CVDs needs to be further summarized and expanded. Finally, therapy targeting ncRNAs or the UPS alone is not yet mature, and cross-linking CVD therapy with the two seems far off.

On the one hand, ncRNA-based therapy offers the advantage of targeting traditionally undruggable targets and a potential role in biomarker-guided therapy. However, there are key challenges to overcome in developing effective ncRNA therapies, such as (1) obtaining sufficient stability and delivery *in vivo*, (2) acquiring high specificity of therapeutic ncRNA for molecular targets, and (3) attaining the ability to target intended cell types or tissues via appropriate vectors and transcriptional targeting and minimizing the side effects of the therapeutic ncRNA itself or drug delivery systems. To reduce these shortcomings, lipid NPs and viral vectors have been widely studied and serve as novel vectors for the delivery of nucleic acid drugs. On the other hand, proteasome, ubiquitin ligase E3 inhibitors and deubiquitinating enzyme inhibitors are undergoing clinical trials and have promising roles in CVD treatment. Nevertheless, proteasome inhibitors may cause selective downstream cell death, the mechanism of which is unclear. The effectiveness of proteasome inhibitor treatment is impaired by innate or acquired resistance mechanisms.

In addition to basic research on signaling pathways, the specific crosstalk of ubiquitin ligases and ncRNAs in CVDs needs to be further investigated with epidemiological evidence and clinical trials. We believe that advanced therapy based on ncRNAs and ubiquitination or deubiquitination can be more effective in treating CVDs and reducing the high mortality rate of CVD for the benefit of all humankind.

## Author contributions

J-RY: Writing – original draft, Writing – review & editing. Z-JW: Writing – original draft, Writing – review & editing. J-WT: Investigation, Writing – review & editing. X-BL: Investigation, Writing – review & editing. RL: Investigation, Writing – review & editing. S-PL: Investigation, Writing – review & editing. HX: Funding acquisition, Writing – review & editing. P-FL: Funding acquisition, Writing – review & editing. Y-FZ: Funding acquisition, Investigation, Writing – review & editing. RZ: Investigation, Writing – review & editing.

## Glossary

**Table:**

CVDs	Cardiovascular diseases
I/R injury	ischemia/reperfusion
A/R injury	anoxia/reoxygenation
H/R injury	hypoxia/reoxygenation
MI	myocardial infarction
HF	heart failure
HT	hypertension
PAH	pulmonary arterial hypertension
AV disease	calcified aortic valve
MCAO	middle cerebral artery occlusion
OGD	oxygen glucose deprivation
UPS	ubiquitin&ndash;proteasome system
E1s	ubiquitin-activating enzymes
E2s	ubiquitin-conjugating enzymes
E3s	and ubiquitin ligases
SUMO	ubiquitin-like modifiers
UBE3A	ubiquitin protein ligase E3a
Ube2i	ubiquitin-onjugating enzyme E2I
UBE2C	ubiquitin E2 ligase C
SUMO1	small ubiquitin-like modifier 1
UCHL1	ubiquitin carboxyl-terminal hydrolase isozyme L1
USP3	ubiquitin specific protease 3
USP7	ubiquitin-specific peptidase 7
USP28	ubiquitin-specific protease 28
circ_UBR4	circular RNA ubiquitin protein ligase E3 componentn-recognin 4
circ-CHFR	circRNA E3 ubiquitin-protein ligase
circ_HECTD1	circRNAs HECT domain E3 ubiquitin-protein ligase 1
ncRNAs	non-coding RNAs
miRNAs	microRNAs
lncRNAs	long non-coding RNAs
circRNAs	circular RNAs
siRNA	small interfering RNA
lincRNAs	long intergenic noncoding RNAs
ceRNAs	competitive endogenous RNAs
RNAi	RNA interference
ZFP36L2	zinc finger protein 36-like 2
CHIP	carboxyl terminus Hsp70-interacting protein
STAT3	signal transducer and activator of transcription 3
HUVECs	human umbilical vein endothelial cells
VSMCs	vascular smooth muscle cells
PASMCs	pulmonary artery smooth muscle cells
Ang II	angiotensin II
CVB3	coxsackievirus B3
NPs	biocompatible nanoparticles
PE	phenylephrine
SRY-box 9 SOX9	transcription factor sex-determining region Y
